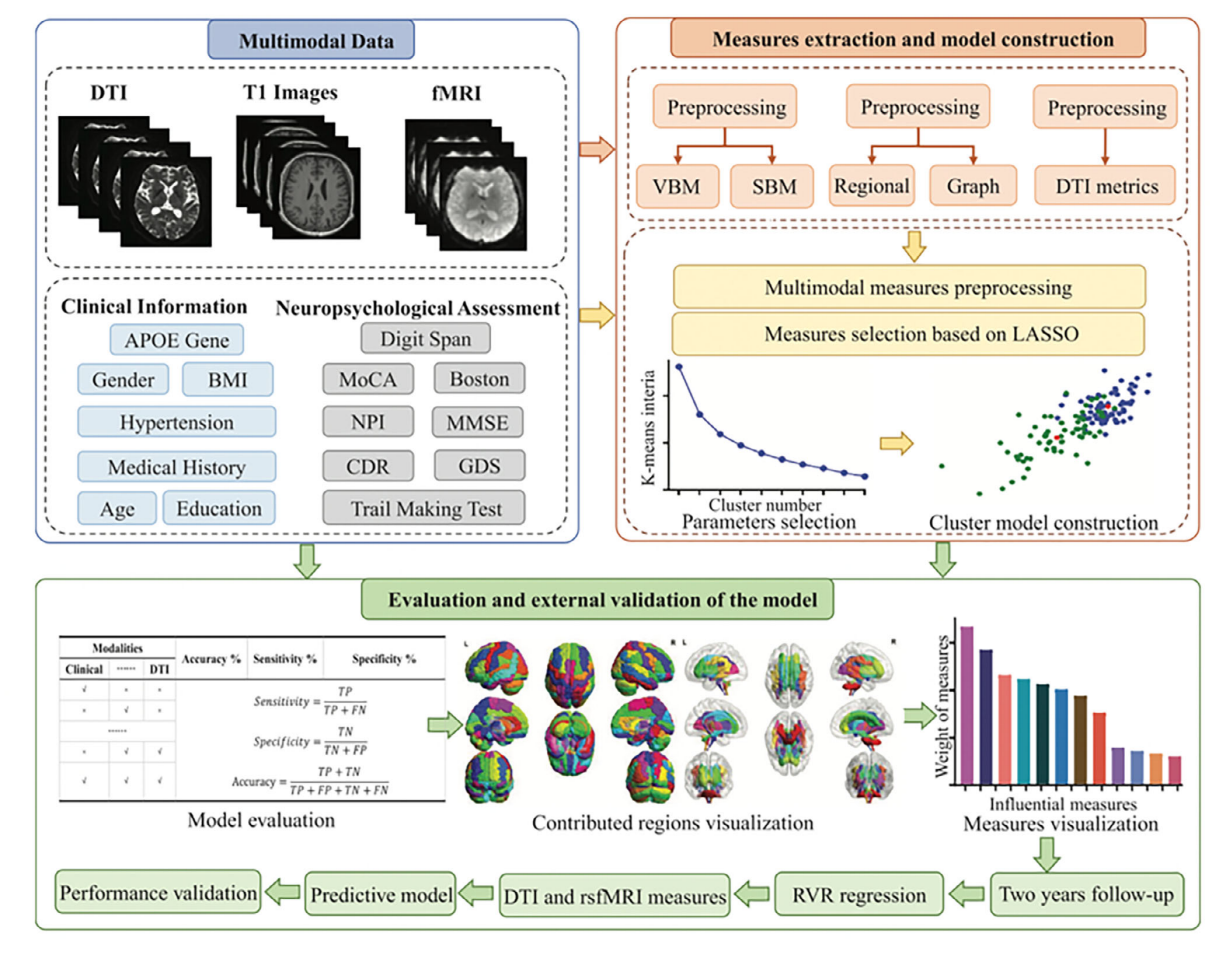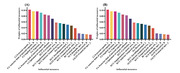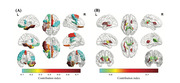# Unsupervised machine learning model to predict cognitive impairment in subcortical ischemic vascular disease

**DOI:** 10.1002/alz.088072

**Published:** 2025-01-09

**Authors:** Qi Qin, Yunsi Yin, Junda Qu, Yi Tang

**Affiliations:** ^1^ Department of Neurology & Innovation Center for Neurological Disorders, Xuanwu Hospital, Capital Medical University, National Center for Neurological Disorders, Beijing, Beijing China; ^2^ Department of Neurology & Innovation Center for Neurological Disorders, Xuanwu Hospital, Capital Medical University, National Center for Neurological Disorders, Beijing, China, Beijing, Beijing China; ^3^ School of Biomedical Engineering, Capital Medical University, 10 Xitoutiao, Youanmenwai, Fengtai District, Beijing, China, Beijing, Beijing China; ^4^ Neurodegenerative Laboratory of Ministry of Education of the People’s Republic of China, Beijing, Beijing China

## Abstract

**Background:**

It is challenging to predict which patients who meet criteria for subcor‐ tical ischemic vascular disease (SIVD) will ultimately progress to subcortical vascular cognitive impairment (SVCI).

**Method:**

We collected clinical information, neuropsychological assessments, T1 imag‐ ing, diffusion tensor imaging, and resting‐state functional magnetic resonance imaging from 83 patients with SVCI and 53 age‐matched patients with SIVD without cogni‐ tive impairment. We built an unsupervised machine learning model to isolate patients with SVCI. The model was validated using multimodal data from an external cohort comprising 45 patients with SVCI and 32 patients with SIVD without cognitive impairment.

**Result:**

The accuracy, sensitivity, and specificity of the unsupervised machine learning model were 86.03%, 79.52%, and 96.23% and 80.52%, 71.11%, and 93.75% for internal and external cohort, respectively.

**Conclusion:**

We developed an accurate and accessible clinical tool which requires only data from routine imaging to predict patients at risk of progressing from SIVD to SVCI.